# Hep88 mAb-initiated paraptosis-like PCD pathway in hepatocellular carcinoma cell line through the binding of mortalin (HSPA9) and alpha-enolase

**DOI:** 10.1186/s12935-014-0069-9

**Published:** 2014-07-22

**Authors:** Panadda Rojpibulstit, Suthathip Kittisenachai, Songchan Puthong, Sirikul Manochantr, Pornpen Gamnarai, Sarawut Jitrapakdee, Sittiruk Roytrakul

**Affiliations:** 1 Faculty of Medicine, Thammasat University (Rangsit Campus), Khlong Luang 12121, Pathum Thani, Thailand; 2 Thailand National Center for Genetic Engineering and Biotechnology (BIOTEC), Thailand Science Park, Phahonyothin Road, Khlong Luang 12120, Pathum Thani, Thailand; 3 Antibody Production Research Unit, Institute of Biotechnology and Genetic Engineering, Chulalongkorn University, Pathum Wan 10330, Bangkok, Thailand; 4 Molecular Metabolism Research Group, Department of Biochemistry, Faculty of Science, Mahidol University, Bangkok 10400, Thailand; 5 Department of Preclinical Science, Faculty of Medicine, Thammasat University, Pathum Thani 12120, Thailand

**Keywords:** Alpha-enolase, Hepatocellular carcinoma, Monoclonal antibody, Mortalin (HSPA9), Paraptosis-like program cell death, Transmission electron microscopy

## Abstract

**Background:**

Hepatocellular carcinoma (HCC) is the most prevalent hepatic cancer worldwide. Currently, a targeted therapy via monoclonal antibodies (mAbs) specific to tumor-associated antigen is undergoing continual development in HCC treatment.

**Methods:**

In this regard, after establishing and consequently exploring Hep88 mAb’s tumoricidal effect on hepatocellular carcinoma cell line (HepG2 cell line), the Hep88 mAb’s specific antigens from both membrane and cytoplasmic fractions of HepG2 cell line were identified by 2-D gel electrophoresis and western blot analysis. After in-gel digestion and subsequent analysis by liquid chromatography-mass spectrometry (LC-MS), mortalin (HSPA9) and alpha-enolase were identified. The recombinant proteins specific to Hep88 mAb were cloned and expressed in *E. coli* BL21(DE3). Moreover, alteration of HepG2 and Chang liver cell line after being induced by Hep88 mAb for 1–3 days was investigated using a transmission electron microscope.

**Results:**

The result demonstrated that Hep88 mAb can bind to the recombinant mortalin (HSPA9) and alpha-enolase. In addition, the gradual appearing of mitochondria vacuolization and endoplasmic reticulum dilatation were observed. Those characteristics might be explained by the paraptosis-like program cell death (PCD), which is induced by the binding of Hep88 mAb to mortalin (HSPA9). Mortalin depletion resulting from the formation of Hep88 mAb-mortalin (HSPA9) complex might initiate transcription-independence of p53-mediated apoptosis. Additionally, Hep88mAb-alpha-enolase complex might initiate HepG2 cells energy exhaustion by glycolysis pathway obstruction.

**Conclusion:**

These fascinating results imply that Hep88 mAb might be a promising tool for the development of an effective treatment of HCC in the next decade.

## Background

Hepatocellular carcinoma (HCC) is the second-most leading cause of cancer-related mortality in the world, accounting for up to approximately 698,000 deaths in 2008 globally [1]. Its prevalence and mortality rate varies from region to region, and is noticeably lower in most developed areas (5–10 per 100,000), but is detected to be gradually higher in developing countries (>20 per 100,000), including the South-East Asian countries [2]. In Thailand, the third leading new cancer cases in males and the fifth in females [3]-[5] are attributed to HCC, whereas the mortality rates rank it as the top leading cancer [6],[7]. The main etiology for HCC in Thailand is hepatitis B virus (HBV) infection [8],[9], while, globally principle etiologies for HCC consist of the chronic infection of hepatitis B virus (HBV), hepatitis C virus (HCV), alcohol and aflatoxin ingestion [10],[11].

Currently, technologies in treatment of the HCC are being continuously developed and include hepatic resection and those of non-surgical management, i.e. percutaneous ethanol injection: PEI [12], transcatheter oily chemoembolization: TOCE [13], radiofrequency ablation (RFA) [14]. However, the mortality rates of the HCC patients are still increasing annually. The survival rates depend on many factors, but especially on tumor size and staging [15]-[17]. This trend is being caused by a lack of sensitive and specific early detection, and effective treatment for the cure of any remaining cancer cells. Alternatively, a targeted therapy such as the immunotherapy via monoclonal antibodies (mAbs) specific to tumor-associated antigen has become a fascinating tool for the targeting of specific HCC cells that are critical to tumor progression while reducing toxicity to normal cells [18]-[20]. The possible mechanisms of anti-tumor effect of mAbs might be involved in blocking the growth-factors receptor, inducing apoptosis or activating complement cascade resulting in tumor cell lysis and phagocytosis by macrophage [21]-[23].

With the advantages of the mAb therapy, we previously established and characterized the cytotoxic activity of Hep88 mAb, a novel monoclonal antibody that fights against HCC [24]-[26]. Our prior studies showed that Hep88 mAb has tumoricidal activity against HCC (HepG2 cell line), while harmless to the normal liver cell line (Chang liver) [25],[26]. Additionally, recognizing proteins against Hep88 mAb have been found not only on cell membranes, but also on the cytoplasmic compartment. In addition, from our previous study, intracellular vacuolization, as well as endoplasmic reticulum and mitochondria dilatation, have been noticed after a 3-day incubated HepG2 with Hep88 mAb [26]. These ultra-structural changes induced HCC death via paraptosis-like program cell death (PCD) [27],[28]. So, we then hypothesized that the killing effect of Hep88 mAb through a paraptosis-like PCD pathway is triggered by binding with its-specific proteins.

Therefore, in this study, Hep88 mAb’s specific antigens were analyzed by 2D-gel electrophoresis and western blot followed by LC-MS. Two specific proteins including mortalin (HSPA9) and alpha-enolase were identified. The cDNA encoding these 2 proteins isolated from HepG2 cells were cloned into the expression plasmid pET17b and expressed in *E. coli* BL21(DE3). Their specific recognitions as Hep88 mAb are rechecked by the western blotting technique. Moreover, the tumoricidal effect of Hep88 mAb against the HepG2 and Chang liver cell line during a 1-3-day period was also monitored using a transmission electron microscope. All of these promising results might lead us to the identification of pharmacological interventions of Hep88 mAb against HCC in the near future.

## Methods

### Cell lines and mAb

Human HCC cell lines, HepG2 cells (American Type Culture Collection [ATCC] HB8065), and normal liver cell line, Chang liver (American Type Culture Collection [ATCC] CCL-13) were cultured in RPMI 1640, supplemented with 10% fetal bovine serum (Biochrom AG, Germany). Both cell lines were maintained at 37°C in a CO_2_ incubator and subcultured every 3–4 days. Hep88 mAb, the anti-HCC mouse mAb, was produced as previously described [24].

### 2D-Gel electrophoresis

Total proteins from cytoplasmic and membranous parts of 4.5 × 10^8^ cells of HepG2 cell lines were extracted, cleaned up and separated by two-dimensional gel electrophoresis as previously described by using immobilized pH gradient (IPG) strips with pI 3–10 and broadening IPG strip with pI 4–7, followed by 12.5% SDS-PAGE [29].

### Western blotting analysis

The gels were subsequently transferred to nitrocellulose membrane (Amersham Pharmacia Biotech Co.) using a semi-dry transblot technique at 6 V, 70 minutes as described by Towbin [30] and Burnette [31]. After blocking the transferred membrane in TBST blocking solution (containing 20 mM TBS pH 7.5, 5% skim milk and 0.1% Tween-20), the membrane was then incubated for 2 hours with Hep88 mAb (dilution 1:2,000) as the primary antibody at room temperature. Thereafter, the membrane was 3× washed in TBST and 1-hour incubated with 1:3,000 alkaline phosphatase-Rabbit Anti-Mouse IgG (H + L) Conjugate (ZyMax™ Grade, Invitrogen) at room temperature. The bounded proteins were color-visualized with BCIP/NBT substrate solution (Invitrogen) according to the manufacturer’s instruction.

### Protein identification

The interested protein spots were consequently in-gel trypsin digested and further analyzed by LC-MS. All obtained MS/MS raw data were submitted to online database search using the MASCOT (Matrix Science, U.K.) against NCBI’s database. Search parameters were set as follows: peptide tolerance (0.2 Da), NCBInr database, Homo sapiens (taxonomy), carbamidomethylation of cysteine (fixed modification), and methionine oxidation (variable modification).

### Plasmids construction of mortalin (HSPA9) and alpha-enolase

Total RNA was extracted from HepG2 cell line by TRIzol reagent (Invitrogen) according to the manufacturer’s instruction. Ten micrograms of total RNA were then used to synthesize cDNA with random hexamers (Invitrogen) and oligo (dT) primers (Invitrogen) by using Superscript reverse transcriptase (Invitrogen). Human *mortalin* (NCBI Reference Sequence: NP_004125.3) and a*lpha enolase* isoform [Homo sapiens] (NCBI Reference Sequence: NP_001419) were amplified by the polymerase chain reaction (PCR). The PCR product of these two proteins were performed with *Bam*HI-NdeI fragment restriction site and cloned in pBluescript (Stratagene). The forward primer sequence for mortalin (HSPA9) was 5′-AAGCTT*CATATG*ATAAGTGCCAGCCGAGCTGCA-3’ and the reverse primer was 5′-*GGATCC*TTACTGTTTTTCCTCCTTTTGATCTTCCTT-3’, while the sequence for α-enolase was 5′-AAGCTT*CATATG*TCTATTCTCAAGATGCATGCCAG-3’ with 5′-*GGATCC*TTACTTGGCCAAGGGGTTTCTGAAGTTC-3’ as the reverse primer. The italic DNA sequence represented an NdeI site on forward primer and a *Bam*HI site on reverse primer, while the bold alphabet ATG on forward primer and the bold TTA on reverse primer referred to start and stop codon, respectively. PCR products were subsequently subject to preliminary analysis on submarine agarose gel electrophoresis and the DNA fragment was purified by using the Nucleospin extraction kit (Nucleospin). After resolving the purified DNA fragment in double-distilled water, the purified DNA fragment was cloned into pGEM-T Easy plasmid and transformed into *E. coli* DH5 alpha. The recombinant plasmid harboring HSPA9 and α-enolase were selected for colonies PCR screening after 37°C overnight incubation on LB/ampicillin plates. The recombinant plasmids were extracted and double-digested with *Bam*HI-NdeI. The nucleotide sequences of the recombinant plasmid containing HSPA9 or α-enolase DNA fragment flanked with *Bam*HI-NdeI restriction sites were determined (Macrogen DNA sequencing Service, Korea).

### Proteins expression

The plasmid containing a corrected sequence of HSPA9 or α-enolase were ligated to an expression vector, pET17b (Novagen) at *Bam*HI-NdeI cloning sites. The recombinant plasmid in pET17b was then transformed into *E. coli* BL21(DE3) and incubated at 37°C overnight on LB/ampicillin plates. To express these 2 proteins, the *E. coli* carrying HSPA9 or α-enolase genes were cultivated at 37°C overnight in LB/ampicillin broth. The overnight culture was diluted 10-fold into fresh LB/ampicillin medium and grown at 37°C until reaching a lag phase (at 0.6-0.7 OD600). Isopropyl-β-D-thiogalactopyranoside (IPTG) at a final concentration of 1 mM was then used to induce protein expression. Cells were harvested after induction for 6 hours at 30°C. Total protein extracted from induced recombinant cells were extracted and analyzed by 10% SDS-PAGE. After running at 100 volts for 2 hours, the gel was visualized by staining with 0.3% Coomassie blue R250.

### Sequential ultrastructure examination by a transmission electron microscope

HepG2 and Chang liver cell lines (1 × 10^6^ cells) were cultured in a completed medium with or without Hep88 mAb at IC50 concentration (12.5 μg/mL) for 0, 1, 2 and 3 days. The preparation procedures for the transmission electron microscope examination, including cell harvesting, glutaraldehyde/paraformaldehyde fixation, osmium post fixation, graded step alcohol dehydration and resin polymerization, were done as previously described [26]. Subsequently, 60–90 nm ultrathin sections were performed by a Porter-Blum MT-2 ultramicrotome and investigated under a Philips CM 100 TEM at 80 kV.

## Results

### Hep88 mAb’s membrane specific antigens detected by 2D-PAGE and western blotting analysis

**Figure 1:**
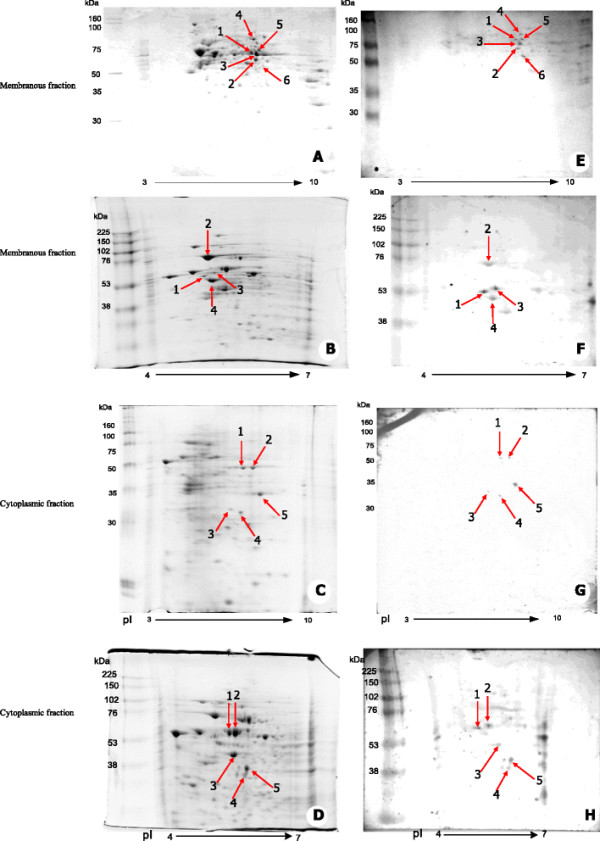
**Proteomic pattern (IEF of pI 3-10 and pI 4-7 followed by 12.5% SDS-PAGE) and immuno-blotting profiles of membranous and cytoplasmic part of HepG2 (1**^**st**^**Ab: Hep88 mAb, dilution 1: 100; 2**^**nd**^**Ab: rabbit anti-mouse Ab, dilution 1: 3,000; Detection system: alkaline phosphatase & BCIP/NBT). (A-B)** Coomassie blue staining of 2-D gel of membrane proteins with IEF of pI 3-10 and pI 4-7, respectively. **(C-D)** Coomassie blue staining of 2-D gel of cytoplasmic proteins with pI 3-10 and 4-7, respectively. **(E-F)** Immuno-staining of membrane proteins with Hep88 mAbwith pI 3-10 and 4-7, respectively. **(G-H)** Immuno-staining of cytoplasmic proteins with Hep88 mAb with pI 3-10 and 4-7, respectively. Protein spots which were further analyzed by LC-MS were indicatedby red arrows.

**Figure 2:**
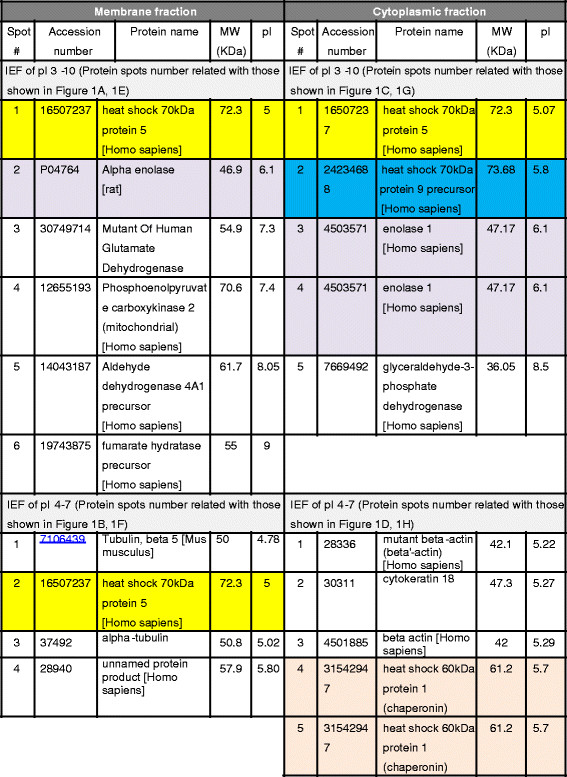
Identified proteins from membrane and Cytoplasmic fractions of HepG2 cells by LC/MS/MS according to the spot number, Swiss-protein accession number, theoretical pI and its molecular weight.

**Figure 3:**
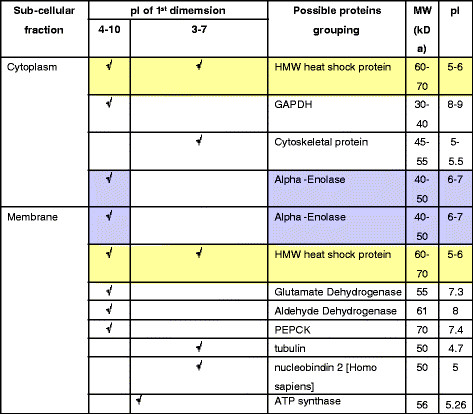
Grouping of the possible proteins from each sub-cellular fraction and each pI in relation with theoretical pI and its molecular weight.

Proteomic study by two-dimensional gel electrophoresis (IEF of pI 3–10 and pI 4–7 followed by 12.5% SDS-PAGE) of membranous and cytoplasmic parts of HepG2 are illustrated in Figure 1A-B and 1C-D, respectively. Protein spots of interest, indicated by the arrows, were visualized after subsequent immunoblotting of membranous and cytoplasmic proteins on 2-D gel with Hep88 mAb, as shown in Figure 1E-F and 1G-H, respectively. LC-MS analysis and peptide sequence comparison with online data base of those protein spots revealed the matched proteins as shown in Figure 2. The summary of matched proteins from both membrane and cytoplasmic fraction in each pI are shown in Figure 3.

### Cloning and expression of recombinant proteins

**Figure 4:**
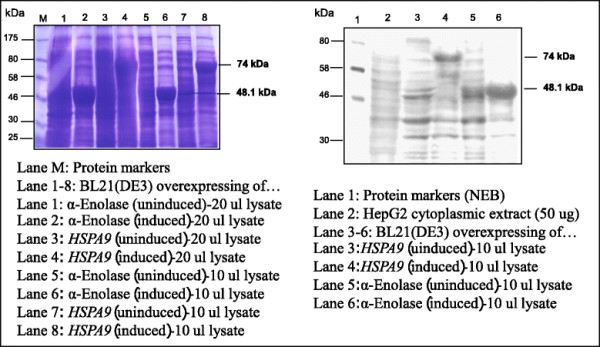
**Expression of** ***HSPA9*** **and** ***α-enolase*** **(Left) Coomassie blue staining (Right) Immuno-blotting staining with Hep88 mAb (1**^**st**^**Ab: Hep88 mAb, dilution 1: 2,000; 2**^**nd**^**Ab: rabbit anti-mouse Ab, dilution 1: 3,000; Detection system: alkaline phosphatase & BCIP/NBT).**

After effectively cloning into pET-17b, and transforming into E. coli BL21 (DE3), the apparent molecular weights of 74 kDa and 48.1 kDa proteins were detected in pET17b-HSPA9 and pET17b-α-enolaseclones, respectively (Figure 4 left). The specificity of those recombinant proteins to Hep88 mAb was then visualized by western blotting technique as shown in Figure 4 right.

### Sequential ultrastructure examination by a transmission electron microscope

**Figure 5:**
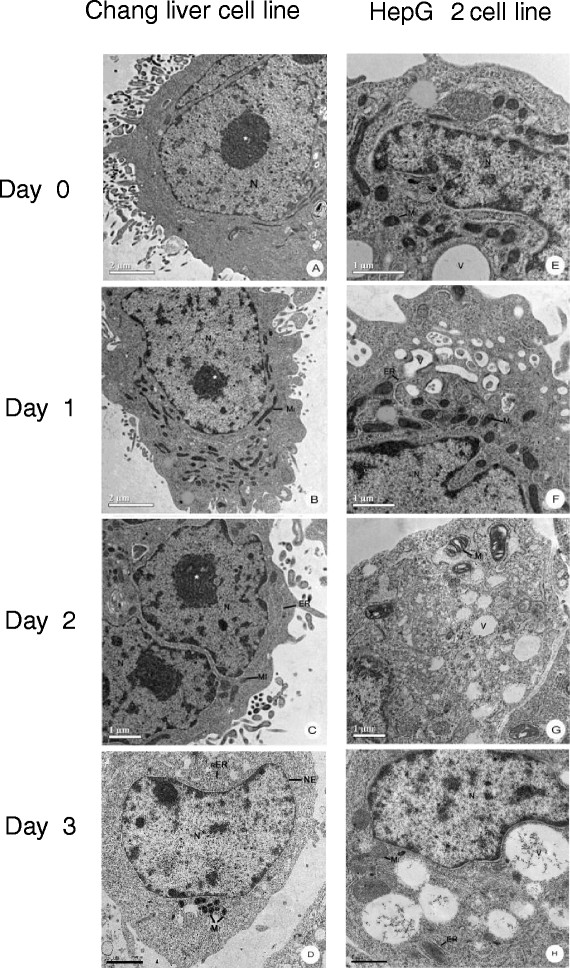
**TEM micrograph of subcellular alterations of the Chang (left) and HepG2 cell line (right) after incubation with Hep88 mAb: day 0 (A, E), day 1 (B, F), day 2 (C, G), day 3 (D, H).** Gradual intracellular vacuolization within mitochondria and ER dilation were observed only in HepG2 treated cell (right).

After incubation the ultrathin section of Chang and HepG2 cell line with the Hep88 mAb and visualization under transmission electron microscope, mitochondria vacuolization and endoplasmic reticulum dilatation were gradually observed on days 0, 1, 2 and 3 only in HepG2 treated with Hep88 mAb as shown in Figure 5.

## Discussion

HCC therapeutic via monoclonal antibody treatments are now being investigated worldwide with the advantages of non-hazardous and non-invasiveness [18]-[20],[32]. In this regard, we previously produced and characterized the new tumoricidal monoclonal against HCC: Hep88 mAb [24]-[26]. By 2D-PAGE and western blot analysis showed that Hep88 mAb recognized both membrane- and cytoplasmic proteins. To confirm the specificity of Hep88 mAb against two protein antigens, the recombinant HSPA9 (mortalin) and α-enolase proteins were expressed in *E. coli* and analyzed by western blot. The ability of Hep88 mAb to trigger the HCC cell death was monitored by a transmission electron microscope. Gradual sub-cellular changes during treatment with Hep88 mAb over a period of 3 days were found.

HSPA9 is a 74 kDa protein, composed of 679 amino acids. It was found both in mitochondria and extra-mitochondrial areas, including cytoplasm and plasma membrane [33],[34]. Because of its multifunction, it was also known as mortalin (an in-cell mortality in mice), mthsp70 (a mitochondrial transport protein in humans) and GRP75 (the glucose response protein in rats) [35]. For cancer modality, it has been proved to be an anti-apoptotic element by its interaction with p53, the tumor-suppressor protein which then inactivates either its transcriptional-dependent or –independent properties and finally diminishes its apoptotic functions. Up to the present moment, the association of over-expressed HSPA9 and HCC has been widely reported [36]-[39]. It is presently said to be a new potential targeted cancer therapy, either by its capacity to disrupt the interaction between GRP75 and p53 or to inhibit its expression [40]-[43].

For the other protein: α-enolase (ENOA), it is a 48 kDa protein, composed of 434 amino acids. It acts as an enzyme catalyzes the reversible reaction of 2-phosphoglycerate (2-PG) to phosphoenolpyruvate (PEP) in a phase II glycolysis pathway. ENOA is the predominant isoform that expressed in almost all adult human tissues. It is also expressed on the cancer cell surface, acting as a plasminogen activator that leads to activate plasminogen into plasmin [44],[45]. This event was further mediated through fibrinolytic consequences resulting in the metastasis of many cancers [46]. For HCC, it has been reported that ENOA over-expression is involved in tumor progression and venous invasion [47],[48]. At this moment, ENOA is a remarkable protein relevant to cancer therapeutic care in a new era [49].

After an expression of recombinant human HSPA9 (mortalin) and α-enolase and subsequently immunoblotting against Hep88 mAb, it was found that both proteins are definitely Hep88 mAb’s specific antigens. From previous reports, up-regulation of HSPA9 (mortalin) was associated with HCC metastasis and recurrence [35],[50]. As is now known, HSPA9 is one of the chaperone proteins whose main functions consist in aiding the folding/unfolding or assembly/disassembly of other multi-proteins that are to be recovered to normal function after stress situation [51]. HSPA9 was found to localize either on plasma membranes, cytoplasmic areas or in mitochondrial parts. Consequently, in cooperation with other proteins, it also functions in concert toward cell proliferation, senescence and tumorigenesis [34],[52]. Wadhwa *et al*. demonstrated that HSPA9 (mortalin) acted as anti-apoptotic factor by preventing p53 activation in cytoplasm [53]. From this view, the ongoing-research is now being pointing out as a striking target for cancer therapy by regaining p53 activity through interfering in the interaction of p53-mortalin complex, as, for example: by its antisense RNA, ribozymes, siRNA or a chemical targeting mortalin-p53 interaction such as MKT-077 (a cationic rhodacyanine dye) [41],[42],[54]. From this point, it has been clearly shown in this present study that Hep88 mAb can react directly with the recombinant human mortalin. In addition, gradual intracellular vacuolization within mitochondria and ER dilation were observed under TEM only in HepG2 after incubation with Hep88 mAb. These subcellular alterations are compatible with those findings in paraptosis-like programmed cell death (PCD) as described by Sperandio *et al*. [27] and Wyllie and Golstein, 2001 [28]. These findings, however, did not match with those as seen in the senescent-arrested cells, i.e. having a large number of electron-dense structures, more lysosomes with a multilayered appearance, as well as an increase in branched or fused mitochondria as reviewed by Terman A *et al*. [55]. Additionally, these alterations also differed from end results found in apoptotic cell death which included DNA fragmentation, membrane blebbing or cytoplasmic shrinkage as summarized by Motyl *et al.*[56]. However, the paraptosis-like PCD which followed the formation of Hep88 mAb-mortalin complex in the cytoplasm might be explained by the releasing of p53 out from p53-mortalin complex, so that the p53 then acted through a transcription-independent pathway by the induction of LMP (lysosomal membrane permeabilization) as discussed by Li *et al.*[57]. This event resulted in the releasing of various lysosomal protease - i.e., granzyme B, cathepsins B and D etc., which finally activated the cells into a state of paraptosis [58].

In addition, the specific recognizable characteristics between α-enolase and Hep88 mAb were also verified in this present study. At this moment in time, α-enolase is believed to be involved in cell migration and cancer metastasis by its action as a plasminogen-binding receptor [44],[45]. Moreover, the correlation between tumor progression and up-regulation of this enzyme has been reported [46]. This finding might be explained by the increased energy demand in cancer cells resulting in forcing the forward feeding of the glycolysis pathway, well-known as the Warburg effect [59]. In this sense, it might be postulated that the effect of Hep88 mAb-α-enolase complex disturbed the Warburg effect and consequently left the cells to die from energy exhaustion. This synergistic effect between the ATP depletion phenomenon along with the effect of the Hep88 mAb-mortalin complex might finally lessen the capacity of the HCC to remain alive.

However, from this study it might be suggested that Hep88 mAb can recognize the share of antigenic epitopes in the structures of HSPA9 (mortalin) and α-enolase, possibly either by their relatively identical 3-D motifs or their similar amino acid sequences at the antigenic determinant, as reported by Piotrowska *et al.*[60]. It is anticipated that this effect will not limit the Hep88 mAb’s efficacy, but, instead, will increase sensitivity of its usage both in therapeutic and diagnosis areas.

## Conclusions

On the basis of these results, Hep88 mAb may well be a new candidate as a monoclonal antibody in the cure of HCC in the near future. However, the existence of its mechanism in the killing of HCC has yet to be proven thus far. But this preliminary study forcefully prods us to learn more about the therapeutic tool of Hep88 mAb.

## Competing interest

The authors declare that they have no competing interests.

## Authors’ contributions

PR contributed in the experimental design, carried out the experiments, analyzed and interpreted the data, and contributed in drafting and revision the manuscript. SK and SR performed the 2D gel electrophoresis and LC-MS analysis. PG carried out the immunoblotting. SP was responsible for Hep88 mAb propagation. SM evaluated the results from an electron micrograph. SJ carried out the cloning and expression of recombinant proteins. All of the authors read the manuscript, contributed in correcting it and approving its final version.
